# Supplementary material for: Effect of fixed 7.5 minutes’ moderate intensity exercise bouts on body composition and blood pressure among sedentary adults with prehypertension in Western-Kenya
Source: PLOS Glob Public Health. 2022 Jul 21;2(7):e0000806. doi: 10.1371/journal.pgph.0000806 (PMC10021634; doi:10.1371/journal.pgph.0000806)
Supplement: S1 Trial protocol — (PDF) [file pgph.0000806.s002.pdf]

The Effect of Fixed 7.5 minutes' Moderate Intensity Exercise bouts on Blood Pressure among Sedentary Prehypertensive Adults in Western-Kenya.

Investigators

Karani Magutah (PhD), Grace Mbuthia (PhD), James Akiruga Amisi (MMED), Diresbachew Haile (PhD), Kihumbu Thairu (PhD)

Background:

Sedentary lifestyles contribute to recent increase in non-communicable diseases in Kenya, with high urbanization and adoption of white collar jobs [1-3]. These diseases have heavy health and economic burden. Among them, hypertension, the commonest cardiovascular disease (CVD) affects 1 in 7 people globally, contributing the most mortality [4-9]. Hypertension, traditionally defined as blood pressure (BP) Ó140/90, has recently included lower values of 130/80. As such, previous values of systolic BP (SBP) 130-139 or diastolic BP (DBP) 80-89 become stage 1 hypertension and values Ó140 or DBP Ó90 are stage 2 [10, 11]. We now know 1 in 3 individuals with higher-normal BP develop hypertension with age [12-14]. Identifying at-higher-risk individuals early and intervening before they convert from pre-disease to full-blown disease is critical [12-15]. This pre-disease state, known as prehypertension, is defined as SBP Ó120-139 and/or DBP Ó80-89 in Ó18 year olds after Ó2 consecutive measurements. Recently, term ñelevatedò BP (SBP 120-129 and DBP <80 mmHg) was proposed, but these proposals by American College of Cardiology and American Heart Association are not yet adopted in Kenya [10-14, 16-19]. Blood pressure data based on this new definition lacks, and available literature is for previous categorization; pre-hypertension. Pre-hypertension affects 25-59% of global population [14, 16]. Independently, it is a modifiable risk factor for CVD but is not a disease itself. The term was coined aiming to identify individuals likely to develop hypertension early, for timely intervention [10, 11, 19, 20].

The large prevalence (26%) of hypertension in sub-Saharan Africa (SSA) poses major social and economic burden, and is a growing concern given its relationship to CVD [6, 21]. A national survey in 2018 showed 25% of Kenyans from have hypertension, and prevalence is similar for rural and urban set-ups, highlighting extent of the burden locally [9, 22].

Prevalence of prehypertension in SSA ranges from 21ï 33% [7, 21]. In 2018, prehypertension prevalence in Kenya was 47% (51% for males; 46% for females) [9], higher than neighbouring Uganda at 33% (42% for males; 29% for females) [23]. Prevalence increases with age peaking at 69 years, then drops thereafter coinciding with increasing hypertension prevalence, suggesting transition of individuals from prehypertension to full-blown hypertension [9]. Thus, the high prevalence of prehypertension, which may lead to hypertension is worrying [9, 12, 13].

Prehypertension is expected to keep rising, posing a grave global challenge. Recently, 20-33% of pre-hypertensive individuals were shown to develop full hypertensive disease within 4 years if no

preventive interventions are employed, and especially if they have higher DBP before age 50. This risk doubles when individuals have prehypertensive BP ranges of 130-139/85-89 mmHg as opposed to 120-129/80-84 mmHg [14, 24-26]. Compared to individuals with BP <120/80 mmHg, prehypertensive individuals have 3-fold higher likelihood of developing full hypertension, yet they are fairly ignored and appropriate management of the problem is vague, underscoring the importance of acting, now [27].

Other than hypertension, pre-hypertension also poses direct risk to CVD by associating with chronic cardiac and vascular changes like arterial stiffness and decreasing intima-media thickness, left ventricular hypertrophy, coronary heart disease, chronic kidney disease and end-stage-renal-disease [12, 28-30]. Modifiable factors such as smoking, being sedentary, obesity, lipidemia, and dietary issues are associated with development of prehypertension, although the path how they contribute in progression from prehypertension to hypertension is unclear [14, 23, 31].

Current recommendations suggest first line management of prehypertension be lifestyle change targeting modifiable factors as opposed to pharmacologic interventions, unless there is concurrent diabetes, kidney, or cardiac disease [9, 16, 19, 27]. In fact, we lack sufficient evidence that pharmacological intervention is beneficial [32]. It has however been long demonstrated that participation in exercise independently lowers BP in both hypertensive and non-hypertensive individuals, and this has strongly been recommended for all [17, 33-35]. Such participation in exercise schedules in younger individuals or those diagnosed with pre-hypertension early remains one of only a few realistic lifestyle-change-ways that could not only lower BP but also mitigate occurrence of full blown hypertensive disease, the others being dietary adjustment and weight loss [12, 16, 36].

Exercise effects on BP vary, some occurring within minutes and others longer term. When exercise is performed for prevention of CVD without drug-treatment, BP gets more hypotensive than that in drug-therapy individuals [37]. Even in resistant hypertension where drug-therapy has failed, aerobic exercise has better results as it has in the other hypertensive cases [38].

Gap in knowledge:

We recently found that exercise regimes involving bouts of <10 minutes but whose cumulative weekly time equals current WHO recommendations of 150 minutes has higher appeal and, yet, confer appreciable health benefits on sedentary normotensive individuals aged Ó50 years [39-41]. Existing guidelines of moderate intensity exercise for adults have traditionally been achieved by performing 30-60 minutes of continuous exercise bouts for 3-5 days weekly, and, for hypertension, there is advocacy to do this daily [35, 41, 42]. Despite our recent find on beneficial health outcomes of shorter bouts, [39, 40], it is unclear if these benefits would translate similarly for sedentary prehypertensive individuals. Longer regimes of Ó30 minutes in 3-5 days weekly as currently recommended are beneficial but lack appeal [35, 43-45]. Studies on optional regimes are scanty, with pockets of emerging data showing accumulating short exercise bouts may impact BP. Emerging knowledge points that accumulating running time of 30 minutes daily, in frequent short bouts of Ó10 minutes, lowers SBP in non-hypertensive individuals in 24 hours to a few days [46, 47]. This, however, not only remains inconclusive, but data on longer term effect of short-bouts in BP lacks. Further, data on effect of short-intermittent exercise on DBP is minimal, but in a study

using 10-minutes-walking exercise tests reduced SBP but not DBP [47]. We are not aware of any randomized-controlled trial that shows what effect sub-10 minutesô moderate intensity exercise would have on sedentary prehypertensive individuals, a neglected subpopulation.

Problem Statement:

Prehypertension prevalence in Kenya today stands at 47%, the highest in SSA [7, 9, 21]. It prevails in males throughout the region (51% and 42% for males versus 46% and 29% for female in Kenya and Uganda respectively) [23]. One in three individuals with such BP develop hypertension and multiple internal organs diseases within four years [12-14, 28-30]. When prehypertension yields hypertension, it poses a major health and economic burden as recently observed in Kenya and other SSA countries [6, 9, 21]. A 2018 Kenyan national survey showed that 25% of individuals of all ages and sex and from rural and urban dwellings have hypertension [22], which prevalence is likely to keep rising as the 47% prehypertensive Kenyans age, thus transitioning to full disease states [9, 12-14].

Yet, there exist lifestyle intervention changes that can revert this trend [14]. Unless we intervene now, that >80% of western Kenyans are sedentary can only enhance heavy transition from prehypertension to full hypertension and CVD [12-15, 48]. Currently, lifestyle change targeting modifiable factors as opposed to pharmacologic interventions is primarily recommended for managing prehypertension [16, 19, 27]. While this includes participation in exercises, long known to lower BP and whose participation guidelines exist for all [41, 49, 50], adherence has been a problem especially when exercise is unsupervised [51]. It is likely that current exercise recommendations lack appeal. This, if so, spells doom for prehypertensive individuals who could otherwise greatly benefit were appealing regimes that raise adherence in exercise available for prescription. Our current proposal therefore hopes to test for more appealing regimes that could be specifically valuable for prehypertensive individuals who would opt for exercise as a way to control their blood pressure, and basing this on guide from our recent find from normotensive persons [39, 40].

Significance:

With high numbers of Kenyans prehypertensive, hypertension prevalence will keep increasing [9, 14]. The cost of screening and treating a hypertensive individual in Kenya is USD 178 monthly [52]. This is worrying in an economy where while 25% are hypertensive and therefore likely to spend heavily on treatment, 36% of them live on <1 USD a day [52, 53]. Thus, a feasible way to prevent transition of prehypertension to hypertension should be sought. Although it is known that participation in physical activity and exercise would help prevent such occurrences, the high sedentariness amongst Kenyans is probably because existing guidelines are not optimally appealing [41, 48]. It is therefore important that to ensure transition to full hypertension is curtailed, other exercise interventions that are intuitively appealing thus raising exercise adherence and so yielding appreciable BP effects be identified. For our set-up, this will reduce numbers developing hypertension, and thus, reduce economic burden associated with treatment of the full disease.

Aim: To evaluate adherence to and BP benefits of cumulative fixed bouts of 7.5-minutesô moderate intensity exercises performed throughout the day as opposed to the single-continuous

30-60 minute bouts among sedentary prehypertensive adults, in quest to mitigate development of full-blown hypertension

Research questions:

1. What is the adherence to fixed bouts of 7.5 minutesô moderate intensity exercises performed thrice daily for 3 months amongst sedentary prehypertensive western-Kenya adults?
2. What is the effect of fixed bouts of 7.5 minutesô moderate intensity exercises performed thrice daily for 3 months on BP amongst sedentary prehypertensive western-Kenya adults?

Objectives:

1. To examine the adherence to fixed bouts of 7.5 minutesô moderate intensity exercises performed thrice daily for 3 months amongst sedentary prehypertensive western-Kenya adults.
2. To assess the effect of fixed bouts of 7.5 minutesô moderate intensity exercises performed thrice daily for 3 months on BP amongst sedentary prehypertensive western-Kenya adults.

Methods:

Design: This will be a randomized controlled field trial amongst residents of Eldoret town, Kenya, where >80% are sedentary [41, 48]. This trial is likely to succeed since our recent work using similar exercise follow-up on an older cohort on different investigations showed improved adherence and had appreciable body composition and cardiometabolic results [54].

Study population and sampling: Shall comprise sedentary adults (Ó18 years, with weekly metabolic equivalent minutes (MET-minutes) <600) using WHO Global PA Questionnaire (GPAQ)). Additionally, they shall be prehypertensive (SBP Ó120-139 and/or DBP Ó80-89). Although BP may vary slightly between sexes, available guidelines do not base on sex, hence recruitment criterion shall be similar. Local print and radio advertisement targeting individuals perceiving themselves as healthy will be made for study volunteers. For motivation, the advert will include that a full physical examination to rule out other health problems will be conducted. Volunteers will be screened to identify 600 participants (3 arms) fitting criteria. ANOVA sample computation using expected DBP means of 72.9±1.4 for participants performing our trial regime (see description below) and 72.2±1.8 for those on the traditional regime as found in our data currently under review for publication, and, 82 mmHg for the non-interventional group expected to maintain baseline values at end point, yields a sample of 100 each for males and females in each of the groups. Being 3 groups, this adds to 600 participants. Considering a drop out estimated at 25%, the sum total sample size will therefore be up to 750 participants. For this computation and using our previous data under consideration for publication, DBP gives larger sample than using SBP. The 750 participants will be proportioned equally through individual-level randomization for each sex into trial arm (7.5-min bouts) T_1, current standard WHO recommendation (Ó30-min bouts) arm T_2, and the non-intervention group T_3 (no guidelines exist for prehypertension care). After signing an informed consent, participants will pick sealed envelopes they personally shuffle, randomly grouping themselves. Thereafter, each shall be explained to what their regime entails.

Protocol description: T_1 participants will perform 3 bouts of 7.5 minutes each daily, their weekly cumulative exercise time reaching 150 minutes. Correspondingly, T_2 participants will perform

current weekly recommendation of 30-60 minutesô sessions for 3-5, similarly yielding 150 minutes. Prescribed shall be home-based moderate intensity exercises (jogging) that raise heart rate (HR) to 50-70% of participantôs maximal expected HR, given as 220 ï (minus) age in years, or where one can talk while jogging but not sing [42]. Activities outside jogging that raise heart rate likewise will also be considered as per attached WHO guidelines in appendix 5. Monitoring for adherence will entail participants wearing polar-wear link accelerometers (GT3X Actigraph, Pensacola, FL, USA) on select days (study cannot afford enough activity monitors) and also by analysing participantsô exercise logs. T_3 will continue normal lifestyle but followed up for BP measurements alongside interventional arms.

Data collection: Baseline data will include bio-demographic characteristics, PA and exercise patterns. HR and BP data before (resting) a run test ï shuttle run test (SRT), at time zero after SRT exertion, and, at 5 minutes rest will also be collected using HR monitors and electronic sphygmomanometers respectively. SRT is an indirect cardiovascular function assessment method utilizing energy expenditure principles. After baseline measurements, T_1 and T_2 participants will perform prescribed exercises from home for 12 weeks. Non-intervention participants will continue normal lifestyles. Home-visits and objective follow-up using accelerometers and participantsô exercise log will monitor progress, providing adherence data. Further, phone reminders will be done for the 12 weeks after which above measurements will be repeated, yielding end-point data.

Analysis: Data will be analysed using STATA v.13 at univariate (BP means and standard deviations) for baseline and week 12, and bivariate level (t-tests; ANOVA) comparing BP data between groups. Multivariate analysis (MANOVA; RM ANOVA) comparing BP between groups and also within groups (repeated measurements) will also be performed. P value Ò0.05 will signify a difference in BP between and within groups.

Ethical consideration: Moi Teaching and Referral Hospital (MTRH) / Moi University Institutional Research Ethics Committee will approve protocol. A physician will remain available throughout implementation phase to handle any adverse effects.

Monitoring and Evaluation

This trial will be considered successful if, at endpoint dropouts in each arm do not exceed 30%, the criterion for follow-ups exceeding 4 weeks [55]. Any arm with higher drop-out will only be considered in answering the adherence but not the effect question. Measures to achieve >70% retention will however be instituted (below). Additionally, a demonstration of mean systolic blood pressure drop by >7 mmHg between baseline and endpoint values will be considered a success, and, further, each intervention arm should have significant BP drop when compared to non-intervention arm.

To achieve this:

- Study personnel will initially be trained on project implementation. Other than study protocols, participants will be trained on ethics of conducting human research. Being an IRB certified researcher myself, I will, together with another IRB member, conduct this. Pre and post training questionnaires will help assess impact of training.

- We will collect weekly exercise data, summarize it, and, where necessary, submit progress reports to IRB in my institution. Any adverse events will be reported within 48 hours and way forward agreed. A data and safety monitoring board (DSMB) will be constituted to ensure continuous monitoring and reporting.
- We will do weekly analysis of exercise logs and accelerometry data to advice on retention of participants or the need to refer for specialized medical attention and thus, discontinuation from protocol for those with enhanced risk.
- We will do phone call and text messaging follow-up, to motivate participants (enhance adherence) and facilitate decision on retention, and, additionally, improve data quality through ensuring accurate observance of prescriptions, to avoid attrition or cross-over effects of adopting non-prescribed exercise regimes.
- We will visit participantsô homes to ascertain protocol adherence and ensure consistency of follow-up by our follow-up team.

Participants whose weekly accelerometry data shows involvement in Ó150 minutes of prescribed exercise and whose BP drops or, at worst, maintains prehypertensive will be retained. Those whose BP rises to hypertensive levels will be reviewed by study physician and referred for pharmacotherapy. Non-intervention group follow-up evaluations will be based on BP changes, retaining only those whose BP does not rise to levels requiring drug-treatment, and referring those who require it.

Expected Results

While all arms will be considered successful if adherence is >70% [55], interventional arm considered as having highest adherence will be that with lowest percentage drop-outs.

To demonstrate whether 7.5-minutesô moderate intensity exercise could lower BP amongst sedentary prehypertensives, expected outcome considered acceptable will be where BP at endpoint is a drop from week 0. At end of follow-up, there must be a significant difference in systolic and/or diastolic BP between baseline and endpoint values for each intervention arm. Similarly, interventional arms must show significant BP change at end-point when compared to non-interventional arm.

Dissemination of research outputs

Dissemination workshops:

a) Participants: Each will get individualized feedback on own BP change. Further, summarised results for all will be shared in workshop, and lifestyle-related suggestions offered to reinforce exercises-adherence long after this study. It is hoped that this will be crucial in participantsô individualized decision making on whether it is important to maintain such exercise in quest to control own BP.
b) Health workers: Seminar with health workers involved in day-to-day management of hypertension will be held, entailing print, overhead projections and interactive discussions on how findings affect prehypertension management. Results will also be shared during regular Continuous Professional Development seminars at our hospital.

c) Uasin Gishu county and MTRH management structures: Workshop for managers from divisions handling non-communicable diseases will be held.
d) General Population: Results will be shared through local-leadership ñbarazaò (Chief meetings where local issues are discussed). A slot during one community *baraza* will be sought. Advocacy for regular BP checks, and, for prehypertensives, adoption of exercise regimes ensuring optimal adherence and BP control will be done.

For scientific audience:

e) Publication: Through open-peer reviewed journals for cardiovascular/hypertension issues.
f) Conference: Findings will be presented in a hypertension and metabolic syndrome conference.

Work plan

**ACTIVITY**	**MONTH -3 to 0**	**MONTH 1 to 3**	**MONTH 4 to 6**	**MONTH 6 to 9**	**MONTH 10 to 12**
Proposal development, designing of tools and mobilization of equipment	X				
Ethical review and approval		X			
Training of research Assistants		X			
Participants recruitment (*screening and consenting*)			X (earlier if approved)		
Data collection (*baseline to endpoint follow-up)*			X	X	
Data analysis and manuscript Writing				X	X
Dissemination of results *(community, conference, seminars, manuscript, health workers)*					X (month 12)

Roles of the study co-PIs

<u>Ms. Grace Mbuthia (BSN, MPH, PhD)</u>

Her clinical expertise will be utilized during screening of participants, randomization, training of data collectors, actual hands-on data collection and co-supervising other data collectors. She will also oversee data entry and cleaning and we together will analyze and write-up our findings.

Being a trained nurse with postgraduate training in epidemiology. She will serve as the co-investigator purely helping out, together with me, in both data collection and study oversight. This is necessary given the various arms of the study, and fact data may be collected from various places and at various points (time), and that will ensure timely completion of the project. She will play crucial role in BP measurements and interpretations both to participants and for the study.

Dr. James Amisi Akiruga (MBChB, MMed)

Dr Akiruga is a family physician who among others deals with hypertension and related diseases. His expertise is needed at recruitment and follow-up, BP interpretation, and the link-referral system for those who may benefit from drug therapy at any point.

As part of the team, he will help in identification of participants, screening for other health issues that may affect participation, and, for those failing inclusion by being hypertensive, not only start their drug-therapy but also provide a link to the health facility where they may continue with management. He will also provide a clinical view on participantsô BP trends and study data.

Dr Diresbachew Haile (PhD)

Being an exercise physiologist, his expertise will be utilised in exercise prescriptions and activity monitoring.

Prof Kihumbu Thairu (PhD)

As an exercise physiologist and also a physician, his twin expertise will help especially in monitoring effect intervention has on individuals, and advise where discontinuation may be necessary.

1. *Ssewanyana, D., et al., Perspectives on Underlying Factors for Unhealthy Diet and Sedentary Lifestyle of Adolescents at a Kenyan Coastal Setting. Front Public Health, 2018.* ***6****: p. 11.*
2. *Ojiambo, R.M., et al., Effect of urbanization on objectively measured physical activity levels, sedentary time, and indices of adiposity in Kenyan adolescents. J Phys Act Health, 2012.* ***9****(1): p. 115-23.*
3. *Onywera, V.O., et al., Emerging evidence of the physical activity transition in Kenya. J Phys Act Health, 2012.* ***9****(4): p. 554-62.*
4. *Poulter, N.R., D. Prabhakaran, and M. Caulfield, Hypertension. Lancet, 2015.* ***386****(9995): p. 801-12.*
5. *Lim, S.S., et al., A comparative risk assessment of burden of disease and injury attributable to 67 risk factors and risk factor clusters in 21 regions, 1990-2010: a systematic analysis for the Global Burden of Disease Study 2010. Lancet, 2012.* ***380****(9859): p. 2224-60.*
6. *Hendriks, M.E., et al., Hypertension in sub-Saharan Africa: cross-sectional surveys in four rural and urban communities. PLoS One, 2012.* ***7****(3): p. e32638.*
7. *Ataklte, F., et al., Burden of undiagnosed hypertension in sub-saharan Africa: a systematic review and meta-analysis. Hypertension, 2015.* ***65****(2): p. 291-8.*
8. *Bromfield, S. and P. Muntner, High blood pressure: the leading global burden of disease risk factor and the need for worldwide prevention programs. Curr Hypertens Rep, 2013.* ***15****(3): p. 134-6.*
9. *Ogola, E., G. Yonga, and K. Njau, A14369 High Burden of Prehypertension in Kenya: Results from the Healthy Heart Africa (HHA) program. Journal of Hypertension. , 2018.* ***36():e330, OCT 2018****.*
10. *Chobanian, A.V., et al., The Seventh Report of the Joint National Committee on Prevention, Detection, Evaluation, and Treatment of High Blood Pressure: the JNC 7 report. JAMA, 2003.* ***289****(19): p. 2560-72.*
11. *Whelton, P.K., et al., 2017 ACC/AHA/AAPA/ABC/ACPM/AGS/APhA/ASH/ASPC/NMA/PCNA Guideline for the Prevention, Detection, Evaluation, and Management of High Blood Pressure in Adults: Executive Summary. A Report of the American College of Cardiology/American Heart Association Task Force on Clinical Practice Guidelines, 2017.*
12. *Redwine, K.M. and B. Falkner, Progression of prehypertension to hypertension in adolescents. Curr Hypertens Rep, 2012.* ***14****(6): p. 619-25.*
13. *Redwine, K.M. and S.R. Daniels, Pre-Hypertension in Adolescents: Risk and Progression. J Clin Hypertens (Greenwich). 2012.* ***14(6): 360-364****.*
14. *Egan, B.M. and S. Stevens-Fabry, Prehypertension--prevalence, health risks, and management strategies. Nat Rev Cardiol, 2015.* ***12****(5): p. 289-300.*
15. *Niiranen, T.J., et al., Prognosis of Prehypertension Without Progression to Hypertension. Circulation, 2017.* ***136****(13): p. 1262-1264.*
16. *Kanegae, H., T. Oikawa, and K. Kario, Should Pre-hypertension Be Treated? Curr Hypertens Rep, 2017.* ***19****(11): p. 91.*
17. *Whelton, P.K., et al., 2017 ACC/AHA/AAPA/ABC/ACPM/AGS/APhA/ASH/ASPC/NMA/PCNA Guideline for the Prevention, Detection, Evaluation, and Management of High Blood Pressure in*

Adults. A Report of the American College of Cardiology/American Heart Association Task Force on Clinical Practice Guidelines, 2018. ***71****(19): p. e127-e248.*
18. *Weber, M.A., et al., Clinical practice guidelines for the management of hypertension in the community: a statement by the American Society of Hypertension and the International Society of Hypertension. J Clin Hypertens (Greenwich), 2014.* ***16****(1): p. 14-26.*
19. *Duprez, D. and A. Toleuova, Prehypertension and the cardiometabolic syndrome: pathological and clinical consequences. Expert Rev Cardiovasc Ther, 2013.* ***11****(12): p. 1725-33.*
20. *Elliott WJ and B. HR., Prehypertension. Nat Clin Pract Cardiovasc Med 2007.* ***4:538-48.***
21. *Guwatudde, D., et al., The burden of hypertension in sub-Saharan Africa: a four-country cross sectional study. BMC Public Health, 2015.* ***15****: p. 1211.*
22. *Mohamed, S.F., et al., Prevalence, awareness, treatment and control of hypertension and their determinants: results from a national survey in Kenya. BMC Public Health, 2018.* ***18****(Suppl 3): p. 1219.*
23. *Nuwaha, F. and G. Musinguzi, Pre-hypertension in Uganda: a cross-sectional study. BMC Cardiovascular Disorders 2013.* ***13:101****.*
24. *Vasan, R.S., et al., Residual lifetime risk for developing hypertension in middle-aged women and men: The Framingham Heart Study. JAMA, 2002.* ***287****(8): p. 1003-10.*
25. *Vasan, R.S., et al., Assessment of frequency of progression to hypertension in non-hypertensive participants in the Framingham Heart Study: a cohort study. Lancet, 2001.* ***358****(9294): p. 1682-6.*
26. *Kanegae H, et al., Which blood pressure measurement, systolic or diastolic, better predicts future hypertension in normotensive young adults? J Clin Hypertens (Greenwich). 2017.* ***19:603-10.***
27. *Preeti G, et al., Prehypertension – Time to Act. Saudi J Kidney Dis Transpl, 2012.* ***23(2):223-233****.*
28. *Huang, Y., et al., Prehypertension and the risk of coronary heart disease in Asian and Western populations: a meta-analysis. J Am Heart Assoc, 2015.* ***4****(2).*
29. *Yuli H, et al., Prehypertension and Incidence of ESRD: A Systematic Review and Meta-analysis. Am J Kidney Dis, 2013.* ***63(1):76-83****.*
30. *Li, Y., et al., A Meta-Analysis on Prehypertension and Chronic Kidney Disease. PLoS One, 2016.* ***11****(6): p. e0156575.*
31. *Song, J., et al., Risk factors for prehypertension and their interactive effect: a cross- sectional survey in China. BMC Cardiovascular Disorders, 2018.* ***18:182****.*
32. *Marta, M., et al., Patients with prehypertension - do we have enough evidence to treat them? Curr Vasc Pharmacol, 2014.* ***12****(4): p. 586-97.*
33. *Whelton SP, et al., Effect of aerobic exercise on blood pressure: a metaanalysis of randomized, controlled trials. Ann Intern Med, 2002.* ***136:493-503.***
34. *Borjesson, M., et al., Physical activity and exercise lower blood pressure in individuals with hypertension: narrative review of 27 RCTs. Br J Sports Med, 2016.* ***50****(6): p. 356-61.*
35. *Pescatello, L.S., et al., Exercise for Hypertension: A Prescription Update Integrating Existing Recommendations with Emerging Research. Curr Hypertens Rep, 2015.* ***17****(11): p. 87.*
36. *Chan J L, et al., The Effects of Diet Alone or in Combination with Exercise in Patients with Prehypertension and Hypertension: a Randomized Controlled Trial. Korean Circ J, 2018.* ***48(7):637-651****.*
37. *Carpio-Rivera, E., et al., Acute Effects of Exercise on Blood Pressure: A Meta-Analytic Investigation. Arq Bras Cardiol, 2016.* ***106****(5): p. 422-33.*
38. *Lopes, S., et al., Exercise as a tool for hypertension and resistant hypertension management: current insights. Integr Blood Press Control, 2018.* ***11****: p. 65-71.*
39. *Magutah, K., et al., Effect of short and long moderate-intensity exercises in modifying cardiometabolic markers in sedentary Kenyans aged 50 years and above. BMJ Open Sport Exerc Med, 2018.* ***4****(1): p. e000316.*

40. Magutah, K., N.B. Patel, and K. Thairu, Effect of moderate-intensity exercise bouts lasting <10 minutes on body composition in sedentary Kenyan adults aged >/=50 years. BMJ Open Sport Exerc Med, 2018. **4**(1): p. e000403.
41. World Health Organization, Global Recommendations on Physical Activity for Health. 2010.
42. CDC, https://www.cdc.gov/physicalactivity/basics/measuring/heartrate.htm accessed on 14/9/2018. 2015.
43. Linke, S.E., L.C. Gallo, and G.J. Norman, Attrition and adherence rates of sustained vs. intermittent exercise interventions. Ann Behav Med, 2011. **42**(2): p. 197-209.
44. Macfarlane, D.J., L.H. Taylor, and T.F. Cuddihy, Very short intermittent vs continuous bouts of activity in sedentary adults. Prev Med, 2006. **43**(4): p. 332-6.
45. Murphy, M.H., S.N. Blair, and E.M. Murtagh, Accumulated versus continuous exercise for health benefit: a review of empirical studies. Sports Med, 2009. **39**(1): p. 29-43.
46. Miyashita, M., S.F. Burns, and D.J. Stensel, Accumulating short bouts of running reduces resting blood pressure in young normotensive/pre-hypertensive men. J Sports Sci, 2011. **29**(14): p. 1473-82.
47. Park, S., L. Rink, and J. Wallace, Accumulation of physical activity: blood pressure reduction between 10-min walking sessions. J Hum Hypertens, 2008. **22**(7): p. 475-82.
48. Nambakai, J.E., et al., Factors influencing participation in physical exercise by the elderly in Eldoret West District, Kenya. African Journalfor Physical, Health Education, Recreation and Dance (AJPHERD), 2011. **Vol. 17, No.3,**: p. 462-472.
49. Garber, C.E., et al., American College of Sports Medicine position stand. Quantity and quality of exercise for developing and maintaining cardiorespiratory, musculoskeletal, and neuromotor fitness in apparently healthy adults: guidance for prescribing exercise. Med Sci Sports Exerc, 2011. **43**(7): p. 1334-59.
50. Physical Activity Guidelines Advisory Committee (PAGAC), Physical Activity Guidelines Advisory Committee Report, 2008. Washington, DC, US Department of Health and Human Services, 2008.
51. Picorelli, A.M., et al., Adherence to exercise programs for older people is influenced by program characteristics and personal factors: a systematic review. J Physiother, 2014. **60**(3): p. 151-6.
52. Oti, S.O., et al., Outcomes and costs of implementing a community-based intervention for hypertension in an urban slum in Kenya. Bull World Health Organ, 2016. **94**(7): p. 501-9.
53. report, W.b., https://data.worldbank.org/country/kenya; accessed on 12/2/2019.
54. Magutah K, Patel NB, and Thairu K, Majority of Elderly Sedentary Kenyans Show Unfavorable Body Composition and Cardio-Metabolic Fitness. J Aging Sci 4:160. , 2016.
55. Furlan, A.D., et al., 2015 Updated Method Guideline for Systematic Reviews in the Cochrane Back and Neck Group. Spine (Phila Pa 1976), 2015. **40**(21): p. 1660-73.

Appendix 1: Informed Consent Form

STUDY TITLE: The Effect of Fixed 7.5 minutes' Moderate Intensity Exercise bouts on Blood Pressure among Sedentary Prehypertensive Adults in Western-Kenya.

Investigators

Karani Magutah (BSN, MPH, MSc, PhD), Grace Mbuthia (BSN, MPH, PhD), James Akiruga Amisi (MBChB, MMED), Diresbachew Haile (PhD) and Kihumbu Thairu (PhD)

Purpose and background:

The study is primarily aimed at assessing the effect of fixed time of moderate intensity exercise involvement amongst individuals aged above 18 years whose blood pressure (BP) measurement is confirmed to be in the prehypetensive ranges. It is anticipated that such exercises will regulate BP and reduce chances of developing full-blown hypertension in such individuals.

Procedure:

If you consent to, you will be interviewed, your cardiovascular function tests of BP and pulse rate done following which you will have an exercise prescription and followed for 3 months. Assessment of your endurance will be done using a field test (shuttle run test)

Benefits:

The major anticipated benefit from this work is that it may advice scientific policy on exercise prescription for individuals with prehypertension. Directly to the participating individuals, they will get to know and monitor their own BP through the 3 months and see how it changes, advising them on value of these exercises in their own BP management

Risk:

There is no known /anticipated direct risk to the participants in the study. However, maximal exertion may cause syncope or other forms of discomfort but a qualified doctor and nurse will be available during your test.

Confidentiality:

All information and measurements will be considered confidential, and consent forms used locked to prevent loss of confidentiality to participants.

Right to refuse or withdraw:

Your participation in the study is entirely voluntary and you are free to refuse to take part or withdraw at any time.

If you consent, please indicate so by signing (or allowing a thumb print) this form:

I agree to participate in this study: …………………… DATE:…………………………..

Lead Investigator (Dr karani Magutah ………………………. Date……………………………..

Appendix 2: <u>Study Participants Advertisement</u>

(IREC Approval Number XXXXXXXX).

We seek to recruit healthy volunteers aged above 18 to a follow-up study on the **"Effect of Fixed 7.5 minutes' Moderate Intensity Exercise bouts on Blood Pressure among Sedentary Prehypertensive Adults in Western-Kenya".**

The study will be conducted within the MTRH and School of Medicine, Moi University.

Participants will undergo a complete physical examination and various cardiovascular function tests before being randomized into either of three groups to receive different exercise prescriptions or none.

This study is expected to last 3 months and will entail close follow-up in participation of the prescribed exercise protocols before end point measurements of similar cardiovascular functions after 12 weeks

This may be intensive.

The study however has benefits apart from advancing knowledge on the exact effect of a 7.5 minutes' fixed exercise prescription for individuals at risk of hypertension: You will know your be able to monitor the effect of these exercise on you own BP and how this changes over 12 weeks.

Volunteers are requested to get in touch with Dr Magutah @ 0721 545 063.

Thank you

Appendix 3: Participants' Record Form

Study number ___________________________

Sex.................

YOBé é é é é

Residenceé é é é é é .

Number of years lived hereé é é é é é .

Occupation..........................

Level of education é é é é é é é é é ..

Physical ailment/injury (if any) ....

Height: é é é é é é cm

Weight: é é é .. kg BMI: é é é é .. kg/m^2

Baseline BP: é é é ..over é é é é ..mmHg (Average of two measurements 5 minutes apart)

Baseline Pulse rate: é é é é .B/M

Last known date (year) of planned/programmed exercise é é é é é

Last known date (year) of hard physical labour/activities .é é é é é

Baseline Height: é é é é é é cm Weight: é é é .. kg BMI: é é é é .. kg/m^2

After 12 weeks Weighté é é é .Kg BMI:é é é é é kg/m^2

Blood Pressure (Note: All measures conducted after five minutes of continued rest ï sitting)

Week 0 (Baseline)

(1st é é é ..over é é é é ..mmHg; 2ndé é é é .overé é é .é .mmHg)

After 12 wks (baseline i.e before test)

(1st é é é ..over é é é é ..mmHg; 2ndé é é é .overé é é .é .mmHg)

Estimated Maximal Heart Rate (MHR) 220-Age = é é é é é é é ..

Heart rate: (Note: All measures conducted after five minutes of continued rest ï sitting)

Week 0 (Baseline) (before test) é é é é .B/M

After 12 weeks (before test) é é é é .B/M

Waist-hip-ratio

Baseline week 0 é é é é é é .. [Waisté é é é é ..cm; Hipé é é é é ..cm]

Week 12 é é é é é é é [Waisté é é é é ..cm; Hipé é é é é ..cm]

Waist-Height-ratio

Baseline week 0 é é é é é é .. [Waisté é é é é ..cm; Hté é é é é ..cm]

Week 12 é é é é é é é [Waisté é é é é ..cm;

Time spent on (*SRT) / level reached

Baseline week 0 é é é é é é ..

Week 12 é é é é é é é

HR at exhaustion/discontinuation from test

week0é é é é .é é é é week12 é é é é .é é é é ..B/M

Estimated VO_{2max} (Relative)

Week 0 test é é é é é é .. mm/kg/min

Week 12 test é é é é é é .. mm/kg/min

Recordings during the Abscissa (Rest Period)

Week 0

Note: BP and HR to be measured only at exhaustion and after 5 minutes of rest.

Time (mins after discontinuation from protocol)	BP	PR
At exhaustion		
At 300 s		

Week 12

Note: BP and HR to be measured only at exhaustion and after 5 minutes of rest.

Time (mins after discontinuation from protocol)	BP	PR
At exhaustion		
At 300 s		

Appendix 4a: <u>Home-Exercise Checklist – 7.5 minutes' bouts</u>:

Typical Weekly Workouts Completed (Adapted from WHO GPAQ questionnaire and showcard):

Jogged (the mandatory prescription for all) or participated in any other activities under Moderate Physical Activity or exercise as per attached GPAQ generic showcard:

for 7.5 minutes 3 times on Monday	Yes	NO	Others (Specify)
for 7.5 minutes 3 times on Tuesday	Yes	NO	Others (Specify)
for 7.5 minutes 3 times on Wednesday	Yes	NO	Others (Specify)
for 7.5 minutes 3 times on Thursday	Yes	NO	Others (Specify)
for 7.5 minutes 3 times on Friday	Yes	NO	Others (Specify)
for 7.5 minutes 3 times on Saturday	Yes	NO	Others (Specify)
for 7.5 minutes 3 times on Sunday	Yes	NO	Others (Specify)

The same protocol shall be maintained for each of the 12 weeks

**At 12 weeks, repeat SRT (as at the baseline) will be done, with the various related cardiovascular measures (methods) before and after the protocol (recovery period) also determined.

Appendix 4b: <u>Home-Exercise Checklist – Traditional bouts</u>:

Typical Weekly Workouts Completed (Adapted from WHO GPAQ questionnaire and showcard):

Jogged (the mandatory prescription for all) or participated in any other activities under Moderate Physical Activity or exercise as per attached GPAQ generic showcard:

Note: This need only be done for 30-60 minutes three to five (3-5) times in a week

for between 30-60 minutes on Monday	Yes	NO	Others (Specify)
for between 30-60 minutes on Tuesday	Yes	NO	Others (Specify)
for between 30-60 minutes on Wednesday	Yes	NO	Others (Specify)
for between 30-60 minutes on Thursday	Yes	NO	Others (Specify)
for between 30-60 minutes on Friday	Yes	NO	Others (Specify)
for between 30-60 minutes on Saturday	Yes	NO	Others (Specify)
for between 30-60 minutes on Sunday	Yes	NO	Others (Specify)

The same protocol shall be maintained for each of the 12 weeks

At 12 weeks, repeat SRT (as at the baseline) will be done, with the various related cardiovascular measures (methods) before, during at after the protocol (recovery period) also determined.

Appendix 5: Global Physical Activity Questionnaire (GPAQ) and Show Card.

**Physical Activity**			
Next I am going to ask you about the time you spend doing different types of physical activity in a typical week. Please answer these questions even if you do not consider yourself to be a physically active person.<br>Think first about the time you spend doing work. Think of work as the things that you have to do such as paid or unpaid work, study/training, household chores, harvesting food/crops, fishing or hunting for food, seeking employment. *[Insert other examples if needed].* In answering the following questions 'vigorous-intensity activities' are activities that require hard physical effort and cause large increases in breathing or heart rate, 'moderate-intensity activities' are activities that require moderate physical effort and cause small increases in breathing or heart rate.			
**Questions**		**Response**	**Code**
**Activity at work**			
1	Does your work involve vigorous-intensity activity that causes large increases in breathing or heart rate like *[carrying or lifting heavy loads, digging or construction work*] for at least 10 minutes continuously?<br>*[INSERT EXAMPLES] (USE SHOWCARD)*	Yes 1<br>No 2 *If No, go to P 4*	P1
2	In a typical week, on how many days do you do vigorous-intensity activities as part of your work?	Number of days └─┘	P2
3	How much time do you spend doing vigorous-intensity activities at work on a typical day?	Hours : minutes └─┴─┘ : └─┴─┘ hrs mins	P3 (a-b)
4	Does your work involve moderate-intensity activity that causes small increases in breathing or heart rate such as brisk walking *[or carrying light loads*] for at least 10 minutes continuously?<br>*[INSERT EXAMPLES] (USE SHOWCARD)*	Yes 1<br>No 2 *If No, go to P 7*	P4
5	In a typical week, on how many days do you do moderate-intensity activities as part of your work?	Number of days └─┘	P5
6	How much time do you spend doing moderate-intensity activities at work on a typical day?	Hours : minutes └─┴─┘ : └─┴─┘ hrs mins	P6 (a-b)
**Travel to and from places**			
The next questions exclude the physical activities at work that you have already mentioned.<br>Now I would like to ask you about the usual way you travel to and from places. For example to work, for shopping, to market, to place of worship. [insert other examples if needed]			
7	Do you walk or use a bicycle (*pedal cycle*) for at least 10 minutes continuously to get to and from places?	Yes 1<br>No 2 *If No, go to P 10*	P7
8	In a typical week, on how many days do you walk or bicycle for at least 10 minutes continuously to get to and from places?	Number of days └─┘	P8
9	How much time do you spend walking or bicycling for travel on a typical day?	Hours : minutes └─┴─┘ : └─┴─┘ hrs mins	P9 (a-b)
10	Do you do any vigorous-intensity sports, fitness or recreational (*leisure*) activities that cause large increases in breathing or heart rate like [*running or football,]* for at least 10 minutes continuously?<br>*[INSERT EXAMPLES] (USE SHOWCARD)*	Yes 1<br>No 2 *If No, go to P 13*	P10
11	In a typical week, on how many days do you do vigorous-intensity sports, fitness or recreational (*leisure*) activities?	Number of days └─┘	P11

12	How much time do you spend doing vigorous-intensity sports, fitness or recreational activities on a typical day?	Hours : minutes └─┴─┘ : └─┴─┘ hrs mins	P12 (a-b)

13	Do you do any moderate-intensity sports, fitness or recreational *(leisure)* activities that causes a small increase in breathing or heart rate such as brisk walking,(*cycling, swimming, volleyball*)for at least 10 minutes continuously? *[INSERT EXAMPLES] (USE SHOWCARD)*	Yes 1<br>No 2 *If No, go to P16*	P13
14	In a typical week, on how many days do you do moderate-intensity sports, fitness or recreational (*leisure*) activities?	Number of days └─┘	P14
15	How much time do you spend doing moderate-intensity sports, fitness or recreational (*leisure*) activities on a typical day?	Hours : minutes └─┴─┘ : └─┴─┘ hrs mins	P15 (a-b)
	**Sedentary behavior**		
The following question is about sitting or reclining at work, at home, getting to and from places, or with friends including time spent [sitting at a desk, sitting with friends, travelling in car, bus, train, reading, playing cards or watching television], but do not include time spent sleeping. *[INSERT EXAMPLES] (USE SHOWCARD)*			
16	How much time do you usually spend sitting or reclining on a typical day?	Hours : minutes └─┴─┘ : └─┴─┘ hrs min s	P16 (a-b)
