# Supplementary material for: Effect of fixed 7.5 minutes’ moderate intensity exercise bouts on body composition and blood pressure among sedentary adults with prehypertension in Western-Kenya
Source: PLOS Glob Public Health. 2022 Jul 21;2(7):e0000806. doi: 10.1371/journal.pgph.0000806 (PMC10021634; doi:10.1371/journal.pgph.0000806)
Supplement: S2 Text — (PDF) [file pgph.0000806.s004.pdf]

REPUBLIC OF KENYA

NATIONAL COMMISSION FOR SCIENCE,TECHNOLOGY & INNOVATION

Ref No: **405214**

Date of Issue: **15/May/2020**

RESEARCH LICENSE

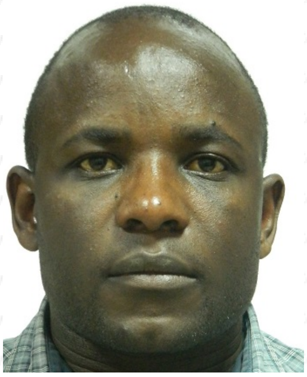

This is to Certify that Dr.. Karani Magutah of Moi University, has been licensed to conduct research in Bungoma, Uasin-Gishu on the topic: The Effect of Fixed 7.5 minutes' Moderate Intensity Exercise bouts on Blood Pressure among Sedentary Prehypertensive Adults in Western-Kenya. for the period ending : 15/May/2021.

License No: **NACOSTI/P/20/4938**

405214

Applicant Identification Number

Director General
NATIONAL COMMISSION FOR SCIENCE,TECHNOLOGY & INNOVATION

Verification QR Code

NOTE: This is a computer generated License. To verify the authenticity of this document, Scan the QR Code using QR scanner application.

National Commision for Science, Technology and Innovation -

THE SCIENCE, TECHNOLOGY AND INNOVATION ACT, 2013

The Grant of Research Licenses is Guided by the Science, Technology and Innovation (Research Licensing) Regulations, 2014

CONDITIONS

1. The License is valid for the proposed research, location and specified period
2. The License any rights thereunder are non-transferable
3. The Licensee shall inform the relevant County Director of Education, County Commissioner and County Governor before commencement of the research
4. Excavation, filming and collection of specimens are subject to further necessary clearence from relevant Government Agencies
5. The License does not give authority to tranfer research materials
6. NACOSTI may monitor and evaluate the licensed research project
7. The Licensee shall submit one hard copy and upload a soft copy of their final report (thesis) within one of completion of the research
8. NACOSTI reserves the right to modify the conditions of the License including cancellation without prior notice

National Commission for Science, Technology and Innovation
off Waiyaki Way, Upper Kabete,
P. O. Box 30623, 00100 Nairobi, KENYA
Land line: 020 4007000, 020 2241349, 020 3310571, 020 8001077
Mobile: 0713 788 787 / 0735 404 245
E-mail: dg@nacosti.go.ke / registry@nacosti.go.ke
Website: www.nacosti.go.ke
