# Supplementary material for: Effect of fixed 7.5 minutes’ moderate intensity exercise bouts on body composition and blood pressure among sedentary adults with prehypertension in Western-Kenya
Source: PLOS Glob Public Health. 2022 Jul 21;2(7):e0000806. doi: 10.1371/journal.pgph.0000806 (PMC10021634; doi:10.1371/journal.pgph.0000806)
Supplement: S3 Text — (PDF) [file pgph.0000806.s005.pdf]

To the Editorial manager

Re: Clinical Trials Registration number

The current study was not registered as a clinical study. We considered it a field study and felt it falls under the exclusion provided in section 801 of the Food and Drug Administration Amendment Act (FDAAA 801) under the statement that "Trials that do not include drug, biological, or device products, such as behavioral interventions" are excluded from mandatory registration in the clinical trials registry. Our study did not involve a drug, a biological or device product but was instead a behavioral intervention in individuals requiring preventive as opposed to therapeutic interventions.

Kindly consider our submission

Karani Magutah

Author

23rd June 2021
