# Supplementary material for: Effect of fixed 7.5 minutes’ moderate intensity exercise bouts on body composition and blood pressure among sedentary adults with prehypertension in Western-Kenya
Source: PLOS Glob Public Health. 2022 Jul 21;2(7):e0000806. doi: 10.1371/journal.pgph.0000806 (PMC10021634; doi:10.1371/journal.pgph.0000806)
Supplement: S4 Text — (PDF) [file pgph.0000806.s006.pdf]

07 July 2021

To Whom It May Concern:

RE: The Effect of Fixed 7.5 minutes' Moderate Intensity Exercise bouts on Blood Pressure and body composition among Sedentary Prehypertensive Adults in Western-Kenya.

As project manager for the Pan African Clinical Trial Registry (www.pactr.org) database, it is my pleasure to inform you that your application to our registry has been accepted. Your unique identification number for the registry is **PACTR202107584701552**.

Please be advised that your trial is registered under an initiative within our system that allow us to capture data of trials that are already in progress or completed. As such, your trial registration may not adhere to the mandates set forth by the International Committee of Medical Journal Editors for registration requirements, and it is your duty to be transparent to any journal that may ask about the retrospective status of your registration.

Please note you are responsible for updating your trial, or for informing us of changes to your trial. Additionally, please provide us with copies of your ethical clearance letters as we must have these on file (via email or post or by uploading online) at your earliest convenience if you have not already done so.

Please do not hesitate to contact us at +27 21 938 0835 or email epienaar@mrc.ac.za should you have any questions.

Yours faithfully,

Elizabeth D Pienaar
www.pactr.org Project Manager
+27 021 938 0835

PACTR
PAN AFRICAN CLINICAL TRIALS REGISTRY
