# Supplementary material for: Effect of fixed 7.5 minutes’ moderate intensity exercise bouts on body composition and blood pressure among sedentary adults with prehypertension in Western-Kenya
Source: PLOS Glob Public Health. 2022 Jul 21;2(7):e0000806. doi: 10.1371/journal.pgph.0000806 (PMC10021634; doi:10.1371/journal.pgph.0000806)
Supplement: S5 Text — (PDF) [file pgph.0000806.s007.pdf]

Reporting Guideline

Section	Item	Page number
Title	The Effect of Fixed 7.5 minutes' Moderate Intensity Exercise bouts on Body Composition and Blood Pressure among Sedentary Adults with Prehypertension in Western-Kenya.	1
Introduction (Background and objectives)	Background and rationale<br>Specific objectives / Aim	2-4<br>4
Methods		
Design	Randomized controlled Field trial	4
Participants	Eligibility: sedentary sedentary (i.e. weekly metabolic equivalent minutes (MET-minutes) <600); and prehypertensive (SBP ≥120-139 mmHg and/or DBP ≥80-89 mmHg)	4
	Setting: Western counties of Kenya	4
Interventions	Short-bout participants performed 3 bouts of 7.5 minutes each of moderate intensity exercises daily; 150 minutes weekly.	4
	Longer-bouts' participants performed 30-60 minutes' sessions of similar intensity for 3-5 days weekly, similarly yielding 150 minutes weekly	4
	The non-interventional group continued normal lifestyles.	5
Outcomes	Exercise adherence	5, 6
		5, 6, 7

	Blood Pressure changes	(and descriptions in tables 1 and 2, and figure 1)
	Change in body composition	5, 6, 7<br>(and descriptions in tables 1 and 2, and figure 1).
Sample size	We studied 665 adults of ages ≥18 years, all volunteers following a local print advertisement	4, 5
Randomization	Individual-level randomization of participants into 3 arms for each sex: trial arm (7.5-min bouts) T_1, current standard WHO recommendation (≥30-min bouts) arm T_2, and the non-intervention group T_3 (no guidelines exist for prehypertension care).	3
Allocation concealment	After signing an informed consent, participants picked sealed envelopes they personally shuffled, randomly grouping themselves.	3
Blinding	Not done	11
Statistical methods	Mean values and their standard deviations.	5,6 and 7<br>Tables 1 and 2
	Between groups' comparisons (ANOVA, repeated measures ANOVA) comparing data between and within groups	5,6 and 7<br>Tables 1 and 2
Results / outcomes	Baseline data	6<br>Table 1

	Endline data	6, 7<br>Table 2<br>Figure 1
Numbers analyzed	Those recording a 12 weeks' adherence to prescribed exercises for all groups (short bouts males n=93, long bouts males n=103, non-intervention males n=78, short bouts females =123, long bouts females n=125, non-intervention females n=96),	7
Discussion<br>Interpretations	In line with results and comparisons with other studies	7-11
Limitations and generalizability	That there was no blinding may have caused peer interactions affecting adherence. Activity monitors were limited for the 665 participants and therefore not available for everyone throughout the follow-up period.	11
Other information<br>Funding source	This research was supported by the Consortium for Advanced Research Training in Africa (CARTA).	11
