# Supplementary material for: Effect of fixed 7.5 minutes’ moderate intensity exercise bouts on body composition and blood pressure among sedentary adults with prehypertension in Western-Kenya
Source: PLOS Glob Public Health. 2022 Jul 21;2(7):e0000806. doi: 10.1371/journal.pgph.0000806 (PMC10021634; doi:10.1371/journal.pgph.0000806)
Supplement: S1 Text — (PDF) [file pgph.0000806.s003.pdf]

INSTITUTIONAL RESEARCH AND ETHICS COMMITTEE (IREC)

MOI TEACHING AND REFERRAL HOSPITAL
P.O. BOX 3
ELDORET
Tel: 33471//2/3

MOI UNIVERSITY
COLLEGE OF HEALTH SCIENCES
P.O. BOX 4606
ELDORET
Tel: 33471/2/3

Reference: IREC/2019/307
Approval Number: 0003551

17th March, 2020

INSTITUTIONAL RESEARCH & ETHICS COMMITTEE
17 MAR 2020
APPROVED
P. O. Box 4606 - 30100 ELDORET

Dr. Karani Magutah & Team,
Moi University,
School of Medicine
P.O. Box 4606-30100,
ELDORET-KENYA.

Dear Dr. Karani & Team,

THE EFFECTS OF FIXED 7.5 MINUTES' MODERATE INTENSITY EXERCISE BOUTS IN BLOOD PRESSURE AMONG SEDENTARY PREHYPERTENSIVE ADULTS IN WESTERN KENYA

This is to inform you that ***MU/MTRH-IREC*** has reviewed and approved your above research proposal. Your application approval number is ***FAN:0003551.*** The approval period is **17th *March, 2020* – *16th March,* 2021.**

This approval is subject to compliance with the following requirements;

i. *Only approved documents including (informed consents, study instruments, MTA) will be used.*
ii. *All changes including (amendments, deviations, and violations) are submitted for review and approval by **MU/MTRH-IREC**.*
iii. *Death and life threatening problems and serious adverse events or unexpected adverse events whether related or unrelated to the study must be reported to **MU/MTRH-IREC** within 72 hours of notification.*
iv. Any changes, anticipated or otherwise that may increase the risks or affected safety or welfare of study participants and others or affect the integrity of the research must be reported to ***MU/MTRH-IREC*** within 72 hours.
v. Clearance for export of biological specimens must be obtained from relevant institutions.
vi. Submission of a request for renewal of approval at least 60 days prior to expiry of the approval period. Attach a comprehensive progress report to support the renewal.
vii. Submission of an executive summary report within 90 days upon completion of the study to ***MU/MTRH-IREC.***

Prior to commencing your study, you will be expected to obtain a research license from National Commission for Science, Technology and Innovation (NACOSTI) https://oris.nacosti.go.ke and also obtain other clearances needed.

Sincerely,

PROF. E. WERE
CHAIRMAN
INSTITUTIONAL RESEARCH AND ETHICS COMMITTEE

cc	CEO	-	MTRH	Dean	-	SOP	Dean	-	SOM
	Principal	-	CHS	Dean	-	SON	Dean	-	SOD
