# Supplementary material for: Effect of fixed 7.5 minutes’ moderate intensity exercise bouts on body composition and blood pressure among sedentary adults with prehypertension in Western-Kenya
Source: PLOS Glob Public Health. 2022 Jul 21;2(7):e0000806. doi: 10.1371/journal.pgph.0000806 (PMC10021634; doi:10.1371/journal.pgph.0000806)
Supplement: Attachment — Submitted filename: Response to Reviewers.docx [file pgph.0000806.s008.docx]

**Editor’s comments**

Comment 1: Restructuring of the abstract

Response: Thank you for the restructured abstract. The original concepts are maintained and we adopt it, with minimal editorials we undertook.

Comment 2: separating description of measurements from data collection sub-heading, and detailing the same.

Response: The measurements done have been described separately from data collection sub-heading and more details provided as suggested

Comment 3: Effect sizes

Response: Thank you for the guidance, we have incorporated the effect sizes where applicable as recommended.

Comment 4: Aspects to consider in discussions

Response: Thank you. We have considered the proposed suggestions and feel this improves our manuscript.

Comment 5: The suggested minor issues have been incorporated in the revision.

**Reviewer 1**

Thank you for your kind observations and comments. We have improved the document following your review.

Specifically, we have worked on the methodology section as recommended, separating data collection and measures done. The various body composition measurements have ben synthesized further. We have clarified that ours was mixed type RMANOVA and, further, included in this revision the effect sizes and F statistics for the regressions performed for the variables associated with the interventions. We have also improved the results and discussions as recommended.

All the “minor” comments have also been noted and addressed, with the exception of an additional table for participants characteristics. We believe that table 1 summarizes the data we had and what we needed to in view of the manuscript. Kindly allow.

**Reviewer 2**

Thank you for your comments. Noted. We have incorporated the suggestions during the edits and improved the manuscript.
