# Supplementary material for: Effect of fixed 7.5 minutes’ moderate intensity exercise bouts on body composition and blood pressure among sedentary adults with prehypertension in Western-Kenya
Source: PLOS Glob Public Health. 2022 Jul 21;2(7):e0000806. doi: 10.1371/journal.pgph.0000806 (PMC10021634; doi:10.1371/journal.pgph.0000806)
Supplement: Attachment — Submitted filename: Responses to Reviewers.docx [file pgph.0000806.s009.docx]

Responses to Reviewers

Reviewer #1:

Sorry for the previous round of responses that were not in a point-by-point approach. Thank you that you read through, we appreciate your kind comments

1. **Comment 1**: “One minor comment is that the manuscript should be thoroughly checked for English language before publication otherwise most of the technical comments were addressed.”

**Response:** We have gone through the entire manuscript checking for language issues and, further, sought help from colleagues with English as their first language.

2. **Comment 2:** “Another comment in the discussion section Paragraph 2, “…adherence in both males and females performing shorter bouts in an older population, proposing that such regimes appeal more…” I suggest the authors to write “adherence in both males and females performing shorter bouts in an older population, suggesting that such regimes may be more appealing”

**Response:** We appreciate the advice and fully adopt it. We have done the change. Thank you

3. **Comment 3** “State data availability at the end of the manuscript”

**Response:** This has been done after the conclusions. Also reflected in legends list.
